# Novel *Mycobacterium* Species in Seahorses with Tail Rot

**DOI:** 10.3201/eid1709.101289

**Published:** 2011-09

**Authors:** José Luis Balcázar, Miquel Planas, José Pintado

**Affiliations:** Author affiliations: Instituto de Investigaciones Marinas, Vigo, Spain (J.L. Balcázar, M. Planas, J. Pintado);; Catalan Institute for Water Research, Girona, Spain (J.L. Balcázar)

**Keywords:** bacteria, Mycobacterium hippocampi sp. nov., polyphasic taxonomic analysis, tail rot, seahorses, letter

**To the Editor:** Seahorses (*Hippocampus guttulatus* and *H. hippocampus*) with signs of tail rot disease (lethargy, lack of appetite, white spots on the skin, and necrotic tail lesions) were collected from aquaria at the Institute of Marine Research, Spain, during March 2007 through May 2009 (Figure A1). Microscopic examination of cutaneous lesions after Ziehl-Neelsen staining disclosed acid-fast bacilli. Microbiologic analysis showed unidentified *Mycobacterium* strains. Subsequently, we used PCR amplification of repetitive bacterial DNA elements to group the strains (*1*). The results showed an identical PCR pattern for the strains; thus, we selected strain BFLP-6^T^ for analysis. On the basis of phenotypic and genotypic data, we consider the unknown acid-fast bacillus to represent a novel species of the genus *Mycobacterium*, for which the name *M. hippocampi* sp. nov. is proposed.

Extraction and amplification of genomic DNA for 16S rRNA sequence analysis were conducted as described (*2*), and the RNA polymerase B (*rpoB*) gene was amplified and sequenced as described by Adékambi et al. (*3*). Sequences obtained were compared against the sequences available in the GenBank, EMBL, and DDBJ databases obtained from the National Center for Biotechnology Information by using the BLAST program (*4*). Phylogenetic analysis were performed by using MEGA version 4.0 (*5*) after multiple alignments of data by ClustalX (*6*). Distances (distance options according to the Kimura 2-parameter model) and clustering with the neighbor-joining method were determined by using bootstrap values for 1,000 replications.

The 16S rRNA sequence of strain BFLP-6^T^ was a continuous stretch of 1,473 bp (GenBank accession no. FN430736). Sequence similarity calculations after a neighbor-joining analysis indicated that the closest relatives of strain BFLP-6^T^ were *M. flavescens* (98.26%), *M. goodii* (98.01%), *M. duvalii* (97.94%), *M. smegmatis* (97.92%), and *M. novocastrense* (97.86%) (Figure). Similar results were obtained for strain BFLP-6^T^ when the maximum-parsimony algorithm was used. The *rpoB* gene has also been proposed as a useful marker for inferring bacterial phylogeny (*7**,**8*). A pair-wise analysis of the *rpoB* sequence of strain BFLP-6^T^ (GenBank accession no. FR775976) showed low levels of similarity (<89.8%) with other species of the genus *Mycobacterium*. The G + C content of DNA, as measured by the thermal denaturation method, was 66.7 mol%.

Strain BFLP-6^T^ was found to consist of gram-positive–staining, aerobic, acid-alcohol–fast, nonmotile, and nonsporulating cells. A scanning electron micrograph showed that strain BFLP-6^T^ is irregular, rod-shaped, ≈1.2–1.4 μm in length, and 0.4 μm in diameter. Colonies on Lowenstein-Jensen medium supplemented with 1.5% (wt/vol) sodium chloride were orange after incubation at 25°C for 5 days. The colonies were positive for catalase, glucose fermentation, arginine dihydrolase, urease, and aesculin, and assimilation of glucose, mannitol, potassium gluconate, and malate. The colonies were negative for nitrate reduction to nitrite, oxidase, indole production, gelatin hydrolysis, *N*-acetyl-d-glucosamine; and assimilation of arabinose, mannose, maltose, caprate, adipate, citrate, and phenylacetate. The major fatty acids were C18:1ω9*c*, C16:0, and C16:1ω6*c*. Mycolic acids included α-mycolates, keto-mycolates, and nonhydroxylated fatty acid methyl esters.

In addition, strain BFLP-6^T^ showed resistance to isoniazid, thiophene-2-carboxylic hydrazide, hydroxylamine, thiacetazone, and picrate. However, the strain exhibited susceptibility to ciprofloxacin, clarithromycin, and rifampin. The type strain BFLP-6^T^ has been deposited in the German Collection of Microorganisms and Cell Cultures, under reference DSM 45391^T^; and in the Belgian Coordinated Collections of Microorganisms under reference LMG 25372^T^.

**Figure:**
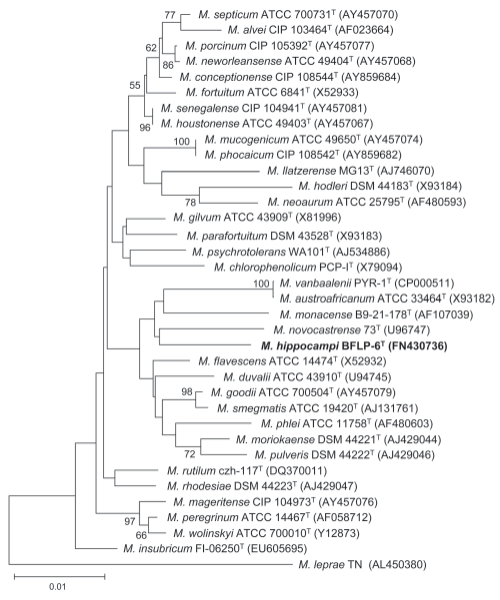
Neighbor-joining phylogenetic tree constructed from 16S rRNA gene sequences, showing the position of strain BFLP-6^T^ (in **boldface**) among other *Mycobacterium* species. Numbers at node indicate bootstrap values (expressed as percentages of 1,000 replications); only values >50% are given. *Mycobacterium leprae* TN was used as an outgroup. Scale bar indicates 0.01 substitutions per nucleotide position. GenBank accession numbers are in parentheses.

## Figures and Tables

**Figure A1:**
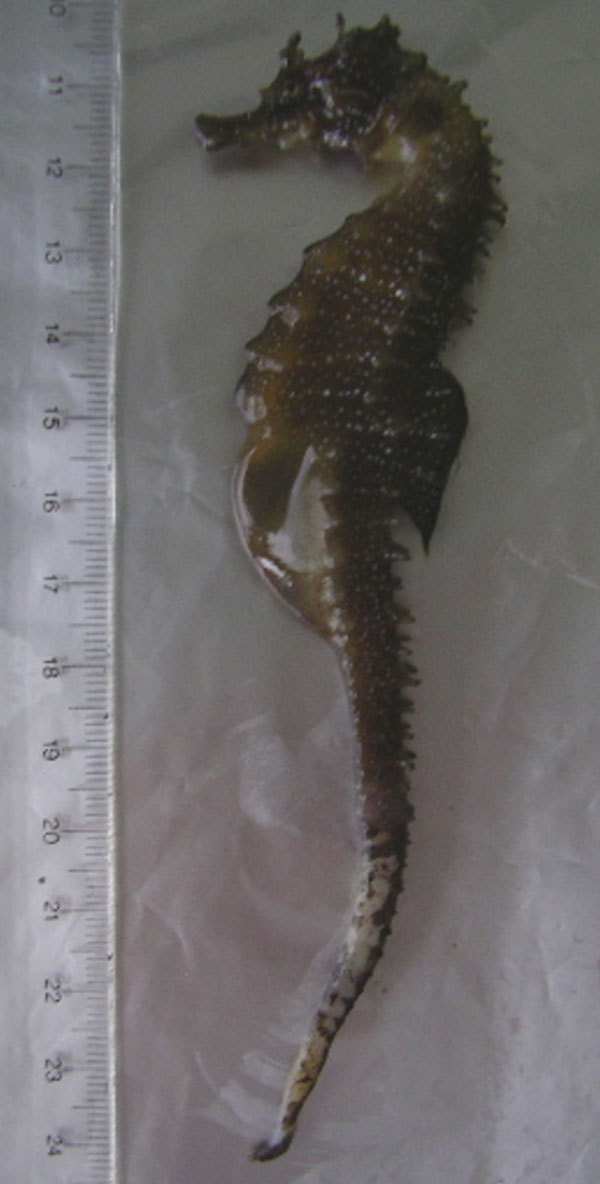
Necrotic tail lesions caused by *Mycobacterium hippocampi* sp. nov. in seahorse (*Hippocampus guttulatus*).
